# Chimeric Antigen Receptor Cell Therapy: Overcoming Obstacles to Battle Cancer

**DOI:** 10.3390/cancers12040842

**Published:** 2020-03-31

**Authors:** Amy J. Petty, Benjamin Heyman, Yiping Yang

**Affiliations:** 1 Department of Pharmacology and Cancer Biology, Duke University, Durham, NC 27710, USA; 2 Division of Regenerative Medicine, Department of Medicine, UC San Diego, La Jolla, CA 92093, USA; 3 Division of Hematology, The Ohio State University, Columbus, OH 43210, USA

**Keywords:** CAR-T cells, CAR-NK cells, CAR-NKT cells, cancer immunotherapy, solid tumors, hematologic malignancies, genetic engineering, novel approaches

## Abstract

Chimeric antigen receptors (CAR) are fusion proteins engineered from antigen recognition, signaling, and costimulatory domains that can be used to reprogram T cells to specifically target tumor cells expressing specific antigens. Current CAR-T cell technology utilizes the patient’s own T cells to stably express CARs and has achieved exciting clinical success in the past few years. However, current CAR-T cell therapy still faces several challenges, including suboptimal persistence and potency, impaired trafficking to solid tumors, local immunosuppression within the tumor microenvironment and intrinsic toxicity associated with CAR-T cells. This review focuses on recent strategies to improve the clinical efficacy of CAR-T cell therapy and other exciting CAR approaches currently under investigation, including CAR natural killer (NK) and NKT cell therapies.

## 1. Introduction

**Figure 1:**
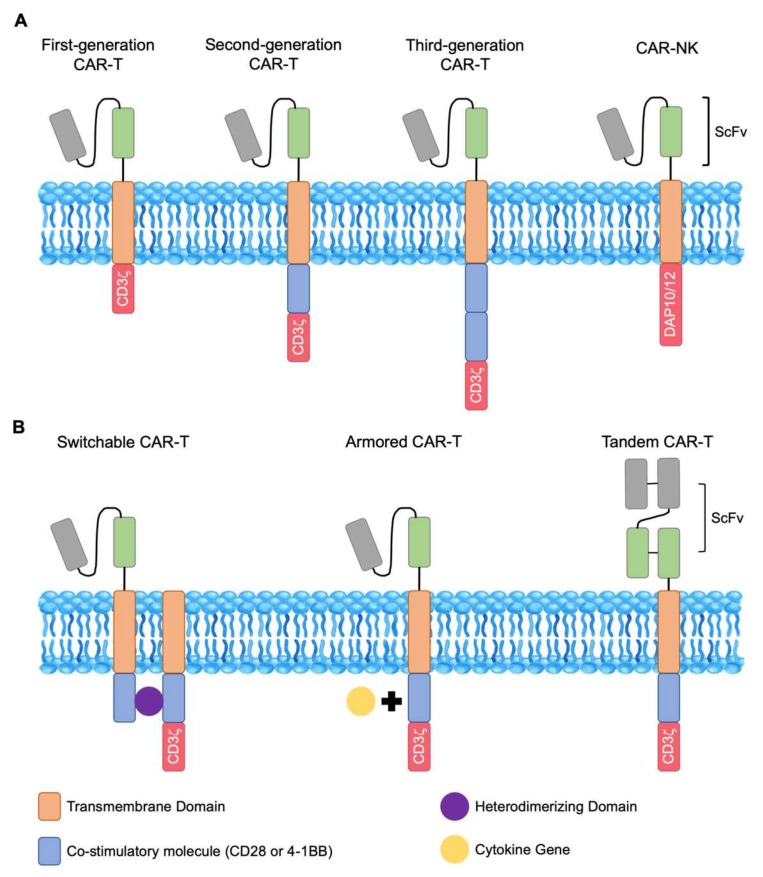
Structures of chimeric antigen receptor (CAR)-T and CAR-natural killer (NK) cells. (**A**) CAR-T cells are engineered by fusing the single-chain variable fragment (scFv) region of a monoclonal antibody to a transmembrane domain and intracellular signaling domains. CAR-NK constructs substitute NK-specific DAP10 or DAP12 protein for CD3ζ. CARs that contain the CD3ζ domain are known as first-generation. Second-generation CARs contain CD3ζ and a co-stimulatory molecule such as CD28 or 4-1BB. Third-generation CARs contain two or more co-stimulatory molecules. (**B**) Switchable CAR-T cells require a heterodimerizing domain as the “on switch” that joins the co-stimulatory domains. Armored CAR-T cells have increased protection against the immunosuppressive tumor microenvironment through the co-expression of cytokines within the CAR-T vector. Tandem CAR-T cells are designed to have two antigen-recognizing domains with one intracellular signaling domain.

Chimeric antigen receptor T (CAR-T) cells-based treatments have made promising advances in cancer immunotherapy. Endowed with the specificity for any cell-surface antigen, these cytotoxic T cells offer potentially curative therapeutic options to patients with previously incurable malignancies. Conceptually, CAR-T therapy requires taking a patient’s native T cells and coupling them with more robust cytotoxic functions. The production of CAR-T cells then requires six steps—apheresis collection from the patient, T cell enrichment, gene modification, activation and ex vivo expansion, quality assessment, and reinfusion into patients. Structurally, a CAR-T cell is a T cell that has been genetically engineered to express an antigen-specific, non-major histocompatibility (MHC)-restricted receptor, which is composed of the single-chain variable fragment (scFv) of an antibody fused to a transmembrane domain and a signaling domain. Initial design of CAR-T cells contained only the CD3-ζ domain, the initiator of T cell receptor signaling intracellularly [1]. These first-generation CAR-T cells revealed limited expansion and persistence in vivo [2,3]. This is due to the fact that successful T cell activation and expansion require both signal one, T cell receptor/CD3-ζ, and signal two, CD28 or 4-1BB, which are costimulatory domains. Therefore, second-generation CAR-T cells were engineered to contain both signals. These improved CAR-T cells demonstrated enhanced cytotoxicity, expansion, and persistence [4,5]. Third-generation CAR-T constructs included both costimulatory domains, with the first being CD28 or 4-1BB and the second being CD28, 4-1BB, or OXO40 (Figure 1A) [6]. Successful CAR construct delivery is usually achieved through electroporation or viral vectors. To date, advances in cell and genetic engineering continued to improve upon previous designs of CAR-T cell constructs. Additionally, strategies to overcome antigen escape and the immunosuppressive tumor microenvironment are under investigation to boost the efficacy of CAR-T therapies. Lastly, modifying natural killer (NK) and natural killer T (NKT) cells with similar strategies have also gained traction in recent clinical trials. In this review, we will focus on recent successes, remaining challenges, and strategies to improve current CAR-based cell therapies.

## 2. Recent Clinical Successes of CAR-T Cells

As of December 2019, there are over 600 active clinical trials along with more than 200 completed trials investigating CAR-T cells around the globe [7]. To date, most clinical investigations of CAR-T cells have focused on hematologic malignancies with CD19-targeting CAR-T therapy demonstrating the most impressive successes. In several early reports, researchers demonstrated up to 70%–90% minimum residual disease-negative complete remissions in patients with advanced, chemotherapy-resistant acute lymphocytic leukemia (ALL) and long term complete remission of about 40% in diffuse large B cell lymphoma (DLBCL) [8,9,10,11,12]. In addition, Kochenderfer et al. found that administration of low-dose cyclophosphamide with fludarabine (Cy/Flu) prior to CAR-T therapy achieved adequate lymphodepletion and that increased serum levels of IL-15 was positively correlated with peak CAR-T expansion [12]. Encouraged by these promising early phase trials, two commercialized CAR-T cell therapies, tisagenlecleucel and axicabtagen-ciloleucel (axi-cel) were put to test in the ELIANA, JULIET, and ZUMA-1 trials [13,14,15]. Tisagenlecleucel is a second-generation anti-CD19 CAR-T cell product that utilizes 4-1BB as a costimulatory domain and lentivirus as a vector. In the international, multi-center phase-II JULIET trial in patients with relapsed/refractory (R/R) DLBCL or transformed follicular lymphoma (FL), tisagenlecleucel treatment resulted in 52% overall response rate (ORR), with a complete response (CR) rate of 40%, a median progression-free survival (PFS) of 2.9 months, and a 49% overall survival (OS) rate at 12 months. Median duration of response (DOR) was not reached during this study. [13]. The efficacy of tisagenlecleucel in pediatric patients and young adults with R/R ALL was also tested in the phase-II, multi-center ELIANA trial which showed an CR of 81%, a PFS of 50%, and OS rate of 76% at 12 months. Remissions were durable with 6-month relapse-free survival of 80% and tisagenlecleucel was detected in patients for up to 20 months [14]. Axi-cel is another second-generation anti-CD19 CAR-T with CD28 as a costimulatory domain. It was studied in patients with R/R DLBCL, transformed FL, and primary mediastinal lymphoma in a large phase-II multi-center ZUMA-1 trial. A total of 108 patients received axi-cel with an ORR of 82%, a CR rate of 58%, a median PFS of 5.8 months, and a 59% OS at 12 months. Median DOR was reported to be 11.1 months (4.2—not estimatable) [15]. As a result, tisagenlecleucel and axi-cel became the first two cellular cancer immunotherapy products that received the US Food and Drug Administration (FDA) and the European Medicines Agency (EMA) approvals in 2017 and 2018, respectively. Following approvals, updated results from the ELIANA trial reported an ongoing response rate of 45% (29 out of 79 patients) and a maximum duration of response of 29 months. For R/R DLBCL patients treated with tisagenlecleucel in the JULIET trial, a median OS for all infused patients was reported to be 11.7 months at the 2018 American Society of Hematology annual meeting [16]. A 2-year follow-up of patients from the ZUMA-1 study proved that responses to axi-cel were durable [17]. Lastly, a third product similar to risagenlecleucel and axi-cel, lisocabtagene-maraceucel (liso-cel) will likely request FDA licensing approval in the near future based on promising results from the TRANCSEND NHL 001 trial [18,19].

Another recent trial used anti-CD19 CAR-T cells in 21 patients with R/R FL and found that CD19 CAR-T immunotherapy was highly effective in adults with clinically aggressive FL with durable remission [20]. In addition to the exciting results from CD19-targeting CAR-T therapies, CAR-T cells targeting other antigens in hematologic malignancies have also reported promising results in recent years. A CD-22 targeting CAR-T therapy induced a 73% (11/15 patients) remission rate in patients with B cell ALL, some of whom were resistant to CD19-targeting CAR-T cells [21]. There have been encouraging results for utilizing CAR-T cells targeting B cell maturation antigen (BCMA) to treat patients with multiple myeloma in small cohorts [22,23]. Moreover, a large multi-center trial of BCMA-CAR demonstrated an impressive ORR of 85% and a CR rate of 45% [24]. Given all the evidence showing that CAR-T therapy is particularly successful in treating bone marrow disease and leukemia, recent efforts have also focused on deploying CAR-T cells in the treatment of acute myeloid leukemia (AML), although progress has been hindered by the lack of leukemia-specific target antigens. Preliminary data from a study of CD123-targeting CAR-T cells for AML patients showed good clinical efficacy without long-term cytopenias. However, results should be interpreted with caution as most patients quickly proceeded to allogeneic hematopoietic stem cell (AHSC) transplantation which eradicated the CAR-T cells [25]. Therapies using CARs to target several other antigens, including CD33, FLT3, NKG2D, CLEC12A, and CLL1 are currently under investigation for AML [26]. Overall, there have been exciting and informative studies in utilizing CAR-T cells in treating patients with hematologic malignancies, particularly ALL and B cell lymphoma. Within B cell lymphomas, most successful clinical outcomes have been observed in R/R DLBCL and FL.

Some recent successes have also been shown in solid tumors though no results from large-scale trials have been reported. In a phase-I dose-escalation trial, Her2-specific CAR-T cells were infused in patients with progressive glioblastoma and found to be safe and clinically efficacious, though no patient achieved complete response [27]. Intracranial infusion of an IL13Rα2-targeting CAR-T product in a patient with recurrent multifocal glioblastoma resulted in regression of all intracranial and spinal tumors, accompanied by increased immune cells in the cerebrospinal fluid [28]. Recently, CAR-T cells against human epithelial growth factor receptor vIII (EGFRvIII), a mutated version of EGFR found in malignant cells, were used to treat 10 patients with R/R glioblastoma and demonstrated good clinical safety profile. One patient had residual stable disease for over 18 months, with all patients showing detectable transient expansion of EGFRvIII-CAR-T cells in peripheral blood [29]. However, the efficacy of CAR-T cells in treating patients with solid tumors are limited despite good preclinical evidence for several antigens, including B7-H3, EGFR, PSCA, CSPG4, and TEM8 [25]. Advances for the use of CAR-T cells in solid tumors will likely rely on further identification of solid tumor-specific antigens, more sophisticated design of CAR-T constructs to enable multiple antigen recognition, and more precise control. 

## 3. Challenges of CAR-T Therapy

Despite exciting progress reported in the last few years for CAR-T therapy, there are a number of challenging obstacles researchers and patients face. For one, toxicity related to therapy is still of concern to clinicians. Additionally, efficacy is limited by non-optimal persistence and potency combined with impaired trafficking to the tumor site in solid tumors. Lastly, tumor heterogeneity and immunosuppression from the tumor microenvironment can further dampen the effects of CAR-T cells.

### 3.1. Toxicity

Major toxicities observed in clinical trials of CARs for the treatment of ALL and B cell lymphoma have been cytokine-release syndrome (CRS), a condition characterized by high levels of inflammatory cytokines that result in a sepsis-like state in patients and CAR-T cell-related encephalopathy syndrome (CRES), a type of neurotoxicity [30]. CRS usually occurs within the first two week of CAR-T infusion and is thought to be related to the upregulation and release of IL-1, IL-2, IL-6, interferon-gamma (IFN-γ), and tumor necrosis factor-alpha (TNFα) from immune cells, especially monocytes/macrophages [31,32,33]. The pathogenesis of CRES is not well understood but has been proposed to be caused by endothelial dysfunctions in the central nervous system (CNS) as a result of highly inflamed state post-infusion [34]. Additionally, neurotoxicity occurs almost exclusively in patients who developed CRS and its severity is positively correlated with higher grade CRS, suggesting some degree of overlap in causative mechanisms [35].

Many factors are thought to be contributive to the development of CRS or CRES in patients, some of which are related to treatment regimen and patient characteristics while others are more intrinsic to the therapy itself. Higher cell doses, expansion, and conditioning chemotherapy with fludarabine have been associated with the development of severe CRS and/or CRES [35,36,37]. In addition, patients with ALL, higher disease burden at treatment initiation and baseline laboratory abnormalities such as thrombocytopenia, elevated angiopoietin-2, and von Willebrand factor are also at a higher risk of developing CRS/CRES [22,23,34,35,36]. Lastly, the structure of the CAR may also explain the difference in toxicity patterns observed as patients treated with CAR-T cell products with a CD28 costimulatory domain were observed to have earlier onset CRS compared to patients treated with 4-1BB containing CAR-T cells [30]. Other trials that utilized CD28-containing CARs reported significant neurotoxicity and death from cerebral edema also raised some safety concerns [12,15,38]. However, to date, there is no definitive evidence showing a direct link between costimulatory domain and toxicity events in patients receiving CAR-T therapy. Review of recent CAR-T trials in patients with ALL also revealed no difference in the rate or grade of CRS and/or CRES with CARs containing CD28 versus 4-1BB costimulatory domains [9,14,37,39]. Lastly, it was suggested that CD19-targeting CAR-T cells were associated with more severe CRES events due to endothelial activation, higher cytokine concentrations in cerebrospinal fluids, and possible crossing of the blood–brain barrier [34]. Further studies are needed to better elucidate the major determinants in CAR-T-related toxicities in hematologic malignancies.

CAR-T-related adverse effects in solid tumor patients are often due to “on-target, off-tumor” effects. In contrast with hematologic malignancies, there is few *tumor-specific* cell-surface antigens and using CARs targeting *tumor-associated* cell-surface antigens could inadvertently damage healthy cells expressing the same antigen. In patients with renal cell carcinoma (RCC) treated with a first-generation CAR-T against carbonic anhydrase IX (CAIX), some patients experienced liver enzyme disturbances that necessitated treatment cessation, a toxicity event that could be eliminated by pretreatment with an anti-CAIX monoclonal antibody [40]. Therefore, better approaches to mitigate toxicity of CAR-T cells are needed.

### 3.2. Sub-Optimal Persistence and Potency

Currently, the degrees of T cell persistent and expansion in vivo are still not optimized, limiting their clinical efficacy, especially in solid tumors [29,41,42,43]. As poor persistence likely contributed to clinical failures observed with CAR-T therapy in solid tumors, several approaches have recently been utilized to improve its persistence, including pretreatment with cytoreductive chemotherapy, optimized T cell culture conditions, and T cell selection procedures. Administration of lymphodepleting chemotherapy containing cyclophosphamide and fludarabine reduced the number of regulatory T cells (T_reg_), which have been shown to negatively impact adoptive T cell transfer [44]. Disappointingly, lymphodepletion in solid tumor patients did not significantly improve the persistence and efficacy of CAR-T cells to the level observed in hematologic malignancies.

In addition to persistence issues, potency of CAR-T cells is limited by T cell exhaustion. This can be induced by excessive stimulation due to high disease burdens and antigen-independent signaling triggered by aggregation of CAR receptors [5,45,46]. Clinically, higher expressions of T cell exhaustion markers on CAR-T cells were found in non-responders when compared to those who achieved complete response in a trial of CD19.BB.z-CAR-T for large B cell lymphoma [47]. Furthermore, expressions of PD-1, TIM-3, and LAG-3 found on T cells pre- and post-engineering were predictive of non-response in CLL patients treated with the same type of CAR-T cells [48]. Collectively, these results suggest that methods that can amplify persistence and potency of CAR-T cells in patients are likely key to treatment success.

### 3.3. Impaired Trafficking

One major obstacle of using CAR-T cells in solid tumors is inefficient localization and infiltration into the tumor stroma. Tissue homing and infiltration require proper expression and precise pairing of adhesion molecules on both the T cells and the vasculature to facilitate leukocyte extravasation towards a chemokine gradient established by tumor cells. However, perfect matching between chemokine receptors on CAR-T cells and the chemokines secreted by tumor cells rarely happen. In addition, recent studies reported reduced chemokine productions as a result of local tumor microenvironment (TME) suppression [49,50]. This can further inhibit CAR-T trafficking to the tumor site. Lastly, aberrant expression of adhesion molecules on the tumor vasculature likely further hindered the accumulation of transferred cells in target tissues [51].

### 3.4. Tumor Heterogeneity

Unlike leukemias and lymphomas, solid tumors often lack specific cell surface markers. Instead, solid tumors are distinguished by anatomic locations, histologic features, molecular mutations, and markers that can be expressed on the surface or intracellularly. Therefore, discovering tumor-specific antigens (TSAs) or tumor-associated antigens (TAAs) that allow for a high-degree of tumor-targeting effects while sparing healthy tissues is one of the most challenging aspects in developing CAR-T cells for solid tumors. Furthermore, finding an ideal antigen that is primarily expressed on the cell surface rather than expressed intracellularly makes the process even more daunting. Though several surface TSAs have been discovered, it was found that there is a great degree of tumor heterogeneity, even among patients suffering from the same type of cancer. Ideally, due to the antigen heterogeneity, it is prerequisite to identify a TSA for each patient and then proceed to generate specific CAR T cells. However, this can be a very complicated engineering process associated with unsustainable high costs for patients and manufacturers. Targeting TAAs, on the other hand can potentially lead to “on-target, off-tumor” effects [52]. Regardless, many TAAs are currently under investigation for the treatment of solid tumors, including CEA, GD2, mesothelin, HER2, MUC1, FAP, LICAM, and IL13Rα [53]. More recently, researchers have increasingly focused on tumor neoantigens that are produced in tumor cells as a result of somatic mutation. However, whether this can be clinically successful is still under investigation. 

### 3.5. Immunosuppressive Tumor Microenvironment

Once at the tumor site, CAR-T cells must also overcome immunosuppressive molecules and cells that could further impede its engagement with a target antigen and/or suppress its cytotoxic functions. In addition, the TME is characterized by harsh conditions, including oxidative stress, nutrient deprivation, acidic pH, and tissue hypoxia, all of which can reduce CAR-T survival and proliferation. Furthermore, tumor cells can upregulate the expression of programmed death ligand 1 (PD-L1) and galectin-9 that are known to suppress T cell functions through checkpoint inhibitory receptors. Lastly, the vast immunosuppressive stromal and immune cell types within the TME, such as cancer-associated fibroblasts (CAFs), tumor endothelial cells (TECs), tumor-associated macrophages (TAMs), tumor-associated neutrophils (TANs), and T_reg_ have all been shown to secrete anti-inflammatory and pro-angiogenic soluble factors to further impede T cell functions [54]. As a result, T cells express high levels of immune checkpoint receptors, including programmed death 1 (PD-1), cytotoxic T-lymphocyte-associated protein 4 (CTLA-4), Lag-3, Tim-3, and Tigit, which are markers of exhaustion associated with reduced effector functions [55]. This combined with all other mechanisms described above can potentially explain the disappointing results observed with CAR-T cells in solid tumors and signal the urgent need to better design CAR-T cells or CAR-T cell combinational therapies to overcome these obstacles.

## 4. Strategies to Improve CARs

To better combat these challenges, additional genetic engineering of CARs combined with synthetic biology and the use of combinational therapy hold the key to provide cellular immunotherapy with novel attributes necessary to overcome its hypo-functionality, trafficking issues, and the immunosuppressive forces in the TME. In addition, efforts to enhance the safety profile of CARs with better spatial and temporal control of their activity and persistence after deployment are also under investigation. The following section highlights some of the recent developments and notable clinical trials are summarized in Table 1.

Recent preclinical and clinical studies reported that decreasing the signal strength of CAR-T cells could lead to diminished toxicity and enhanced persistence. Ying et al. identified a CAR variant, CD19-BBz(86), that produced significantly lower levels of cytokines than the prototype CD19-BBz while retained potent catalytic activity. As a result, a phase I study with 25 patients of B cell lymphoma revealed no significant CRS or CRES events while achieving a CR rate of 54.5% [56]. Others engineered alternative regions of the CAR-T constructs and found that decreased cytokine production was associated with less severe CRS/CRES in patients and better clinical outcomes [57,58]. In addition to creating novel constructs associated with reduced cytokine production, others have developed a “safety switch” or “advanced cell programming technology” in CARs to prevent or limit the likelihood of toxicity. These are termed “*Switchable CARs*” that do not target any specific surface target antigen and only become operational in the presence of a bispecific adaptor molecule that mediates connections between components of the CAR-T construct (Figure 1B) [59,60,61]. In addition to providing a self-limiting safety switch since the CAR-T cells automatically turn off after rapid elimination of the adaptor molecule, these constructs also allow for gradual clearance of cancer cells through in vivo titration, minimizing the risk of acute toxicity in patients with high tumor burden. Lastly, switchable CARs offer better specificity control post-adoptive transfer by delivering adaptor molecules targeting various antigens. This may also be an effective strategy to overcome antigenic loss and tumor heterogeneity.

Faced with non-optimal CAR-T cell persistence after re-infusion, there is growing interest in developing protocols that can better preserve the less-differentiated T cells, such as naïve and central memory T cells since higher percentages of these T cell subsets prior to genetic engineering and infusion were associated with improved in vivo persistence [62]. Ghassemi et al. showed that reducing the duration of ex vivo culturing to 3–5 days yielded less differentiated cells with better therapeutic efficacy compared to cells expanded with the standard 9–12 days protocol [63]. Others substituted IL-7 and IL-15 for IL-2 as the growth factor during ex vivo expansion of CAR-T cells as this cytokine combination was demonstrated to specifically enrich the T memory stem cell population and further showed that CAR-Ts expanded IL-7 and IL-15 have better persistence and antitumor activity [64,65]. More recently, Shum et al. constructed a constitutively signaling cytokine receptor C7R that potently triggers the IL-7 signaling pathway in CAR-T cells and showed superior T cell proliferation, survival, and function in metastatic neuroblastoma and orthotopic glioblastoma xenograft mouse models [66]. Kagoya et al. also created a novel JAK-STAT signaling domain-containing CAR that demonstrated increased proliferation and persistence and decreased terminal differentiation [67]. Mechanistically, it was shown that phosphoinositide 3-kinase (PI3K) activation led to decreased CAR-T cell persistence and inhibition of the PI3K resulted in enhanced antitumor activity [68,69]. Finally, the emergence of “*Armored CARs*” that constitutively secrete cytokines such as IL-12. IL-15 and IL-18 also provided further hope that more sophisticated engineering of CAR-T cells can overcome the persistence issue all together (Figure 1B) [70,71,72,73].

Decreased potency of CAR-T cells can also be prevented by site-directed integration of CAR-T construct that simultaneously eliminates specific genes that are known to suppress T cell functions. Using the CRISPR/Cas9 technology, Eyquem et al. were able to direct a CD19-targeting CAR to the T cell receptor α constant (*TRAC*) locus, resulting in enhanced potency and antitumor activity [74]. Additionally, Fraietta et al. reported a case of CAR-T integration into the Tet methylcytosine dioxygenase (*TET*2) locus, leading to impressive potency of the particular clone in a CLL patient [75]. Lastly, Feucht et al. showed that CARs with each of the three immunoreceptor tyrosine-based activation motifs (ITAMs) can have different cell fates, suggesting fine calibration of CARs to balance replicative capacity of long-lived memory cells with the acquisition of strong effector functions can further improve therapeutic potency [76].

Based on insights from mechanistic studies that showed chemokine receptors CXCR3, CCR2, and CCR5 are critical for T cell trafficking to the tumor site [77], several preclinical studies have investigated strategies that increased chemokine signaling in CAR-Ts. Moon et al. genetically modified CAR-T cells to express CCR2 and showed enhanced trafficking of these cells towards mesothelioma tumors [78]. In addition, genetic deletion of protein kinase A (*PKA*) resulted in increased expression of CXCR3 in CAR-Ts, which was associated with improved targeting to the tumor site and tumor control [79]. A current trial testing CAR-T cells co-transduced with the chemokine receptor CCR4 is currently underway for patients with CD30^+^ non-Hodgkin’s lymphoma (NHL) but more clinical studies are needed to further investigate the therapeutic advantages of chemokine-receptor-expressing CAR-T cells [80]. Another approach to bypass inadequate trafficking to the tumor site involves local or regional delivery of CAR-T cells without additional engineering, though this may not always be technically achievable. In a preclinical study involving mesothelioma, investigators found that intrapleural delivery of anti-mesothelin CAR-T cells led to superior clinical performance with 30-fold fewer cells required to induce long-term complete remission [81]. Early clinical results in patients with thoracic cancers showed that intrapleural administration of CAR-T cells was safe and had promising antitumor activity [82]. Regional delivery of CAR-T cells is particularly appealing to CNS tumors since a previous study has shown delivery into the cerebrospinal fluid is safe [28]. Two recent preclinical studies also highlighted potential efficacy and reduced toxicity when delivering CAR-T cells intraventricularly [83,84]. Currently, there are several clinical trials investigating regional delivery of CAR-T cells in various cancer types, including CNS tumors, hepatocellular carcinoma (HCC), and pancreatic adenocarcinoma [7].

There is a great deal of antigenic heterogeneity in cancer and, as a result, therapies targeting one molecule can rarely achieve complete tumor eradication. In addition, antigenic loss or remodeling has also emerged as one of the major obstacles in CAR-T cell therapy. Currently, several strategies to augment antigen expression targeted by CAR-T cells have achieved success in preclinical settings. Inhibition of gamma-secretase which increased the expression of BCMA or adding all-trans retinoic acid which upregulated CD38 both have found to be beneficial in augmenting CAR-T cell functions in multiple myeloma [24,53]. Additionally, several groups developed “*Tandem CARs*”, which are bispecific CAR-T cells that have two scFvs against two different antigens linked by a single molecule in the same CAR-T construct or bicistronic CARs in which two monospecific CARs are expressed from the same vector (Figure 1B). Preclinical successes using bispecific CAR-T cells have been reported in solid tumors, including the use of a tandem CAR against Her2/IL13Rα2 in a murine glioblastoma model. These tandem CAR-T cells were able to form stronger immune synapses, resulting in more sustained effector function without evidence of exhaustion compared to unispecific CAR-T cells [85]. For R/R ALL and NHL, bispecific CD19/CD20 and CD19/CD22 CARs are currently in clinical trials but preliminary results demonstrated good efficacy with favorable safety profile [86,87,88,89]. Furthermore, epitope-spreading is another power and complementary approach to overcome tumor heterogeneity. Inflammation induced by the CAR-T cells may promote the presentation of neo-antigens to be recognized by native T cells of the host immune system, potentially leading to elimination of CAR-targeted antigen-negative tumor cells. Sampson et al. demonstrated this in a mouse model of glioblastoma using EGFRvIII-targeting CAR-T cells. Infusion of CAR-T cells lead to elimination of both antigen-positive and antigen-negative tumor cells [90]. However, this phenomenon has not been well studied and further investigation is needed to better utilize it to overcome tumor heterogeneity.

One of the most common and successful approaches to boost T cell functions in the immunosuppressive TME is the use of immune checkpoint receptor (ICR) inhibitors, including antibodies against PD-1 and CTLA-4. Co-administration of PD-1/PD-L1 blocking antibodies with CAR-T cells was shown to restore CD28 CAR-T cells’ persistence and cytotoxicity in the TME in a mouse model of mesothelioma. Clinically, using CD19-targeting CAR-T cells combined with anti-PD-L1 antibodies in patients with R/R DLBCL showed promising results with a 90% ORR and six patients in CR [91]. Additionally, an anti-PD-1 antibody, Nivolumab combined with anti-CD19 CAR-T cells also demonstrated impressive clinical efficacy with an ORR of 81.8% and CR of 45.5% in a total of 11 patients with R/R NHL [92]. In children with R/R B cell ALL, preliminary results of combinational therapy with PD-1 blockade and CAR-T cells were promising [93]. Currently, there are two clinical trials investigating whether ICR blockade can augment CAR-T functions in patients R/R classical Hodgkin’s lymphoma (NCT04134325) and glioblastoma (NCT03726515) [7]. Others explored genetic engineering that allows for the deletion of *PD1* in CAR-T cells or in vivo production of anti-PD-1/PD-L1 inhibitors. Several reports highlighted enhanced CAR-T cell activities when *PD1* or *LAG3* were deleted via CRISPR/Cas9 in preclinical studies [94,95,96,97]. An anti-CD19 CAR-T cell that constitutively secretes anti-PD-1 inhibitors was tested in a CD19^+^ lung cancer xenograft model and showed enhanced T cell proliferation, survival, and cytotoxicity [98]. In other studies, researchers using CAR-T cells genetically engineered to secrete anti-PD-L1 or anti-PD-1 scFvs in mouse models of metastatic RCC or ovarian cancer, respectively, also showed superior performance when compared to conventional CAR-Ts or with co-administration of checkpoint inhibitors [99,100]. Currently, CAR-T cells engineered to secrete PD-1, PD-L1, or CTLA-4 antibodies are under investigation in clinical trials for various hematologic and non-hematologic malignancies. In addition to ICR inhibitors, other promising strategies to boost CAR-T functions include the use of immunomodulatory drugs such as lenalidomide or pomalidomide and T cell-stimulating monoclonal antibodies such as daratumumab or isatuximab in multiple myeloma. In preclinical studies, lenalidomide was shown to increase cytokine production and cytolytic activity of CAR-T cells in mouse models [24]. Currently, a clinical trial (NCT03070327) utilizing anti-BCMA CAR-T with or without lenalidomide is underway to examine its efficacy in patients with multiple myeloma [7].

Lastly, dominant negative genes have been successfully introduced into CAR-T cells to overcome immunosuppression in the TME. Kloss et al. engineered prostate membrane antigen (PSMA)-targeting CAR-T cells to co-express a dominant-negative transforming growth factor-beta (TGFβRII) which negates the suppressive effects of TGFβ signaling in T cells. When tested in a mouse model of prostate cancer, investigators found that these TGFβRII-expressing CAR-T cells demonstrated enhanced persistence and potency and are more resistant to exhaustion. A clinical trial investigating its effects in patients with metastatic prostate cancer is currently underway [101]. Recently, Yamamoto et al. genetically engineered CAR-T cells to express a dominant-negative Fas variant, rendering T cells resistant to Fas ligand-induced apoptosis. They discovered that these T cells have enhanced persistence and superior antitumor efficacy against established solid and hematologic cancer [102]. Taken together, these recent approaches proved that next-generation CAR-T cells designed with sophisticated genetic-engineering techniques are poised to improve upon the already impressive clinical progress made in B cell hematologic malignancies and expand therapeutic options into solid tumors. 

## 5. CAR NK and CAR NKT

The role of NK and NKT cells has been studied in cancer. The ability of NK cells to exert cytolytic functions on cancerous cells through the release of perforin and granzyme B (GzmB) as well as through FasL–Fas interactions, combined with its non-MHC-restricted pattern of activation make it an attractive option for cellular immunotherapy [103]. On the other hand, NKT cells represent an important link between the innate and adaptive immune systems and can be activated through antigen-dependent or -independent mechanisms. After being activated by antigen presented by MHC class I-like CD1d molecule, they rapidly produce inflammatory cytokines such as IFN-γ, IL-17, GM-CSF, and TNF-α [104].

Each cell type is an appealing alternative for CAR engineering for different reasons. First, allogeneic NK cells do not cause graft-versus-host disease (GVHD) as shown in clinical studies using haploidentical and cord blood-derived NK cells in patients with hematologic malignancy or solid tumors [105,106]. Secondly, NK cells do not go through massive clonal expansions and have limited lifespan in vivo, thereby reducing the risk of CRS and long-term adverse side effects. Lastly, CAR-NK cells retain their native receptors and it is theoretically less likely for cancer cells to escape surveillance in the event of CAR antigen downregulation [107]. NKTs are of particular interest because of the CD1d restricted nature of NKT antigen recognition, potentially limiting off-target toxicity and increasing applicability in autologous and allogeneic settings [103].

Engineering of CAR-NK cells include the same manufacturing scheme as CAR-T cells and a recent novel approach that substitutes NK-specific intracellular activation domain for CD3-ζ (Figure 1A). This modification using NK-specific DNAX activating protein 10 (DAP10) and 12 (DAP12) showed promising results in preclinical studies with CAR-NK cells demonstrating superior activation and function [108,109]. To date, there are four active clinical trials evaluating the clinical efficacy of CAR-NK infusions in patients with hematologic malignancies and solid tumors—NCT03415100, NCT03056339, NCT03940833, and NCT03940820 (Table 2) [7]. However, many challenges remain to be addressed before CAR-NK therapy can be widely used for larger cohorts of patients. The isolation, expansion, and transduction process for CAR-NK cells are yet to be perfected for better in vivo NK persistence and efficacy. Further studies are definitely needed to explore optimal construct, vector, and transduction methods to make CAR-NKs more useful in cancer immunotherapy.

CAR-NKTs also showed promising results in several recent studies. Heczey et al. engineered CAR-expression NKT cells targeting GD2 in neuroblastoma and CD19 in lymphoma and observed great success in preclinical studies. These CAR-NKT cells not only had high selectivity for target tumor antigens but can also proliferate and produce high levels of cytokines efficiently within the TME [110]. Currently, this study has been translated into a clinical trial evaluating GD2-specific CAR-NKT cells in children with neuroblastoma (NCT03294954) (Table 2) [7]. Additionally, CD19-specific NKT cells were found to be more effective than CD19-targeting CAR-T cells against CD1d-expressing lymphomas in vitro and in vivo, suggesting CAR-NKTs can provide unique advantages in treating lymphomas and other CD1d-expressing tumors [111]. However, the same challenges faced by CAR-NK therapy remain true for CAR-NKT cells and more studies are needed to improve our current understanding and manufacturing scheme of CAR-NKT cells to benefit more patients.

## 6. Conclusions and Perspective

Overall, recent advances made in the field of CAR-based therapy are exciting and impressive. Many patients with hematologic malignancies have greatly benefitted from CAR-T cell treatments. Though there is less success in solid tumors, better engineering techniques, delivery, and combinational therapies may hold the key for improved outcomes in these patients. Lastly, the emergence of other CAR-based cellular therapies, including CAR-NK and CAR-NKT cells shed additional insights and provided hope that advances in bioengineering may enable broader and more effective usage of these immune cells in cancer.

## Figures and Tables

**Table 1:** Clinical trials with novel CAR strategies.

Trials	Disease	Trial Results or Ongoing Studies
**Reducing Toxicities**		
NCT02842138	R/R B cell lymphoma	- Phase I trial utilizing CD19-BBz(86) which produces lower levels of cytokines: CR 54.5%, no CRS or CRES reported.
NCT02443831	R/R ALL andBurkitt lymphoma	- Phase I trial utilizing a lower affinity CAR-T construct: one-year survival 63%, no severe CRS reported.
**Improving Persistence and Potency**		
NCT03274219	R/R Multiple myeloma	- Phase I trial investigating a next-generation anti-BCMA CAR-T cells bb21217 with better persistency and potency.
NCT04093648	R/R HCC	- Phase I trial investigating Glypican-3-targeting CAR-T cells that coexpress IL-21 and IL-15
NCT02498912	R/R MUC16ecto+ solid tumor	- Phase I trial investigating IL-12-secreting CAT-T cells targeting MUC16ecto antigen.
**Increasing Trafficking**		
NCT03602157	R/R HL and CTCL	- Phase I trial of CAR-T cells targeting CD30 antigen and expressing CCR4 for improved trafficking.
NCT02414269	Malignant pleural disease	- Phase I trial of intrapleural administration of mesothelin-targeting CAR-T cells.
**Overcoming Immunosuppression**		
NCT02926833	R/R DLBCL	- Phase I trial combining Axi-Cel with atezolizumab: 90% overall response rate.
NCT03726515	Glioblastoma	- Phase I trial utilizing EGFRvIII-targeting CAR-T cells combined with pembrolizumab
NCT03706326	Advanced esophageal cancer	- Phase I/II trial investigating anti-Muc1 CAR-T cells with PD-1 knockout
NCT03070327	Multiple myeloma	- Phase I trial investigating anti-BCMA CAR-T cells with or without lenalidomide

**Table 2:** Clinical trials involving CAR-NK or CAR-natural killer T (NKT) cells.

Trials	Disease	Trial Results or Ongoing Studies
**CAR-NK Cell Trials**		
NCT03415100	Metastatic solid tumors	- Single arm, open-label pilot study evaluating NKG2DL-targeting CAR-NK cells
NCT03056339	R/R ALL, CLL and NHL	- Phase I/II trial utilizing umbilical cord blood-derived CAR-NK in conjunction with lymphodepleting regimen
NCT03940833	R/R Multiple myeloma	- Phase I/II trial evaluating BCMA-targeting CAR-NK 92 cells
NCT03940820	Solid tumor	- Phase I/II trial investigating ROBO1-targeting CAR-NK cells
**CAR-NKT Cell Trials**		
NCT03294954	R/R Neuroblastoma	- Phase I trial investigating IL-15-secreting CAT-NKT cells targeting GD2-antigen
